# Offspring genes indirectly influence sibling and maternal behavioural strategies over resource share

**DOI:** 10.1098/rspb.2017.1059

**Published:** 2017-09-27

**Authors:** David G. Ashbrook, Naorin Sharmin, Reinmar Hager

**Affiliations:** 1 School of Biological Sciences, Faculty of Biology, Medicine and Health Sciences, University of Manchester, Manchester M13 9PT, UK; 2 Department of Biological Sciences, University of Toronto, Scarborough, Ontario, Canada

**Keywords:** parent–offspring conflict, indirect genetic effects, systems genetics, family conflicts

## Abstract

Family members show behavioural strategies predicted to maximize individual fitness. These behaviours depend directly on genes expressed in focal individuals but also indirectly on genes expressed in other family members. However, how sibling and parental behavioural strategies are modified by genes expressed in family members, and to what degree, remains unclear. To answer this question, we have used a split litter design in an experimental population of genetically variable mouse families, and identified loci that indirectly affected sibling and maternal behaviour simultaneously. These loci map to genomic regions that also show a direct effect on offspring behaviour. Directly and indirectly affected traits were significantly correlated at the phenotypic level, illustrating how indirect effects are caused. Genetic variants in offspring that influence solicitation also impacted their siblings' and maternal behaviour. However, in contrast to predictions from sibling competition, unrelated litter mates benefited from increased solicitation. Overall, such indirect genetic effects explained a large proportion of variation seen in behaviours, with candidate genes involved in metabolism to neuronal development. These results reveal that we need to view behavioural strategies as the result of conjoint selection on genetic variation in all interacting family members.

## 1. Introduction

Although social behaviours are expressed by individuals, their fitness effects depend on the behaviour shown by social partners. Understanding the evolution of social behaviour is a major challenge in biology because trait variation is not just influenced by genes expressed in focal individuals but also by genes expressed in social partners [1]. While theoretical work has shown that this dual genetic control of social traits fundamentally alters predictions about trait evolution [2], we still know little about the actual genes underlying social traits and the importance of their indirect effects [3,4].

For mammals, the most important social interactions occur during early development between parent and offspring, and among siblings [5,6]. However, family members are in conflict over resource share and level of parental investment with offspring favouring greater parental investment than is optimal for the parent, and individual siblings claiming more than their fair share of parental provisioning. Behavioural strategies affecting solicitation, provisioning, and resource share are therefore selected to maximize different fitness optima for different family members [7,8], however, fitness pay-offs are dependent on behaviours shown by all members. Thus, variation in genes expressed in individuals showing a particular behaviour and those expressed in family members will influence the response of that particular behaviour to selection and its evolution.

What remains unclear is the degree to which a particular behavioural strategy (such as the level of offspring solicitation) is influenced by genes expressed in a focal individual (a direct effect) or genes expressed in other family members (an indirect effect). Further, we do not know how genes expressed in one family member affect the behaviour of other members.

**Figure 1.**
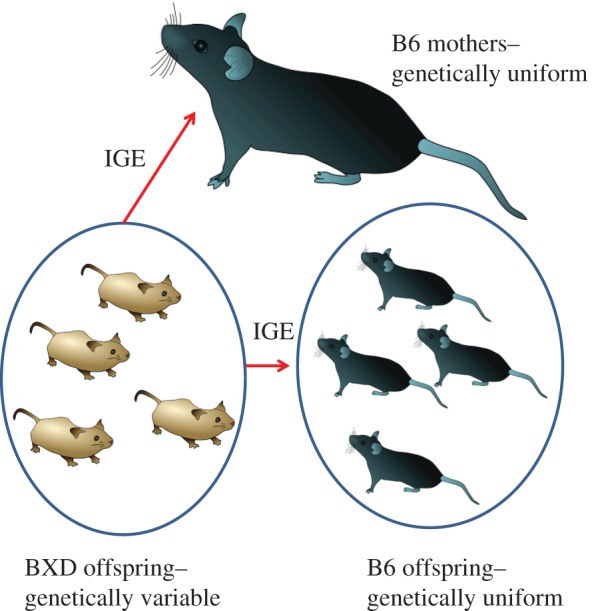
Experimental half-litter cross-fostering design. B6 mothers adopt half litters of different lines of the BXD population and half litters of B6 offspring. Genetic variation among BXD genotypes causes indirect genetic effects (IGE) in both mothers and siblings.

A key problem in answering these fundamental questions is to separate direct from indirect effects on behaviours during social interactions because in a genetically variable population all genotypes are likely to exert both direct and indirect effects. Our previous work [9] has shown that offspring solicitation and maternal behaviour are significantly correlated at the phenotypic level but could not demonstrate how sibling and maternal behaviour is influenced simultaneously by genes showing a direct and indirect effect. We hypothesized that indirect effects can explain a large proportion of variation in behavioural traits in all family members and designed an experiment that enabled us to determine the effects of genetic variation in offspring on traits in unrelated litter mates and unrelated foster mothers. We investigated behavioural interactions in an experimental population of genetically defined mice, in which adoptive families consist of half genetically variable (using the recombinant inbred strain BXD) and half genetically uniform offspring (using the inbred strain C57BL/6 J or B6; figure 1). We recorded in this population, from birth until weaning, maternal provisioning and activity, as well as offspring solicitation, sucking (from maternal teats), and activity, independently in both half litters following [10]. Next, we conducted a quantitative trait locus (QTL) interval mapping analysis that enabled us to locate where in the genome genetic variation causes indirect effects and investigate in more detail potential candidate genes.

## 2. Material and methods

We investigated behavioural interactions among siblings of different genotype and their adoptive mothers on 3 days during lactation, and used single nucleotide polymorphism (SNP) interval mapping to map trait variation in genetically uniform individuals (B6 mothers and B6 offspring) as a function of genetic variation in their (BXD) siblings to investigate indirect genetic effects (IGE). By contrast, direct genetic effects were investigated by mapping behavioural variation among BXD individuals to their own genotype. The QTL analysis was followed by a systems genetics analysis to identify functional candidate genes and biological pathways. We then analysed the phenotypic correlation between the behavioural traits affected by direct and indirect genetic variation. The genetic variants are identified as social interaction loci together with the chromosome number, e.g. *SocInt1* would denote a locus on chromosome 1.

### (a) Experimental animals

Our project used mice of the BXD recombinant inbred population, which consists of experimentally tractable and genetically defined mouse lines capturing a large amount of naturally occurring genetic variation, which underlies variation at the phenotypic level (e.g. [11]). BXD is the largest genetic model system in mammals and consists of over 140 experimentally tractable mouse lines and has the largest phenome of any mammalian model system (5 388 traits, March 2017; greater than 1 k papers since 2003, http://www.genenetwork.org). BXD is derived from two divergent mouse strains (C57BL/6 J and DBA/2 J, hence BXD), in which different recombination patterns have been inbred, each with a fixed recombination pattern of exactly two possible alleles. BXD incorporates 4–5 million segregating single nucleotide polymorphisms, 500 k insertions and deletions, and 55 k copy number variants [12,13].

In this experiment, we used 32 lines (from BXD lines 1, 11, 24, 38, 43–45, 48a, 49, 51, 55, 56, 61, 62, 64, 65a, 65b, 65, 68–71, 73a, 73b, 73–75, 84, 87, 90, 100, and 102), which were selected to exclude very poor breeding lines. For each line, three within-line replicates were set up, although breeding success reduced this in some lines. C57BL/6 J (B6) inbred mice were used as the genetically uniform strain such that all mothers and half of each litter had the same genotype in all cases (figure 1). BXD mice were obtained from Prof. Robert W. Williams at the University of Tennessee Health Science Centre, Memphis, TN, USA, and C57BL/6 J mice were obtained from Charles River, UK. All procedures were approved by the University of Manchester Ethics Committee.

### (b) Husbandry and mating protocol

Mice were maintained under standard laboratory conditions in the same room, exclusively used for the experiment, in individually ventilated cages (IVC Tecniplast Green line), maintained at 20(±2)°C with a relative humidity of 55% (±10%). Because we investigated behavioural patterns in a nocturnal species, we used a reverse dark : light cycle with red light between 10.00 and 22.00 h. Food and water was provided *ad libitum*. Cages were cleaned once a week but never within the first 6 days after birth to minimize disturbance. The parental mice were all sexually mature and females were nulliparous. Groups of up to five sibs were housed together in single-sex cages until mating, which occurred between six and 10 weeks of age, when females were greater than or equal to 18 g. Prior to mating bedding from the prospective mate's cage was added to the female cages to encourage synchronized oestrus [14], and individual males were moved to new cages to allow them to scent mark. Two days later two sisters were added to the male's cage. Once visibly pregnant (weight gain ≥8 g or distended abdomen), females were separated into an individual cage. This ensured that neither father nor aunt had a social interaction with the offspring.

### (c) Experimental design

Females in individual cages were checked daily for new-born litters. Litters were weighed and cross-fostered, such that each B6 mother had a litter composed of half B6 and half BXD offspring (figure 1). On a few occasions (less than 10% of litters) no corresponding litters were available for cross-fostering, in which case the procedure was delayed by a day. If no corresponding litter was available on the following day, the individuals were removed from the study. Cross-fostering after birth meant that all litters were genetically, and as far as possible, environmentally, identical with the exception of the genotype of the BXD pups.

For 6 days following cross-fostering, the litter was left undisturbed, apart from visual checks from outside the cage twice daily, to minimize disturbance.

On postpartum days 6, 10, and 14, observations were conducted following [15]. Litters and mothers were weighed and then separated for 4 h. Mothers were placed in a new cage with the food and water from the original cage while the litter was left in the original cage and placed on a heat mat to keep the pups warm during the separation. This standardizes as much as possible the motivation for maternal care when they are reunited with their pups but also reduces variation in offspring motivation due to differences in care received prior to observations. To distinguish between half litters, we used colour differences where coat colours are different between the respective BXD line and B6. Where this was not the case, we used small fur clippings for one genotype but varied this randomly between B6 and BXD. Maternal and pup behaviour was recorded during the 15 min after the pups were reunited. Behaviours were separated into states (long-lasting, commonly occurring activities) and events (short, less common). States were recorded by scan sampling every 20 s, and events were recorded whenever they occurred. Offspring behaviour was recorded as the number of pups (of an individual genotype) engaged in the behaviour at any given time, and an average for the litter was used for statistical analysis. Offspring solicitation behaviour is defined as pups attempting to suck and following the mother, while sucking refers to the actual feeding behaviour while being attached to teats. Activity refers to active behaviour other than solicitation such as moving around. For all offspring measures, individual pups were not distinguished. For mothers we focused on provisioning behaviour, which is suckling, and other activity, such as digging and moving around.

### (d) Quantitative trait locus mapping and candidate analysis

To account for differences between litters not due to genotype differences, residuals were calculated from a general linear model (GLM) with the following covariates: maternal bodyweight, average bodyweight of the B6 offspring (weight of the B6 litter divided by the B6 litter size), B6 litter size, average bodyweight of BXD offspring (weight of the BXD litter divided by the BXD litter size), BXD litter size, and batch. Non-significant (*p* > 0.05) terms were removed sequentially and in a stepwise manner until only significant covariates remained in the model following [16]. All GLMs were carried out using SPSS (v. 21, IBM Corporation, Armonk, NY, USA).

For QTL analyses, the average trait value per line was calculated and residuals from the GLMs were mapped using interval mapping as implemented in GeneNetwork (GN). Interval mapping relies on 3 795 informative SNP markers across all chromosomes, except Y. The BXD strains were genotyped using the MUGA array in 2011, along with genotypes generated earlier using Affymetrix and Illumina platforms [17], and mm9 was used. Loci are identified in GN by the computation of a likelihood ratio statistic score and significance was determined using 5 000 permutations of the phenotype data. Confidence intervals were given by a LOD drop of 1.5 from the peak marker location [18]. To investigate how indirect effects arise from direct effects, we scanned the genome for co-location of indirect effect QTL and direct effect QTL, where both the direct effect locus and indirect effect locus have to be at the same genomic location (i.e. within the same region as given by the confidence intervals; table 1). Since we analysed four traits at three different time points (overall 24 tests, with just over 1 locus expected to be a false positive) during lactation we have used the false discovery rate (FDR) criterion following [19] that applies a correction for multiple testing based on the number of rejected null hypotheses. For our study, all loci had to be significant at the genome-wide level for either a direct or indirect effect during the genome scan and pass the threshold following [19]. While it is important to protect against many false discoveries, both at the genome-wide level when scanning for one trait and when considering multiple scans, we need, at the same time, assurance we are not ignoring truly interesting effects (e.g. [20]). Thus, where a locus was identified at the genome-wide level and it passed the Benjamini and Hochberg threshold, we then investigated indirect effects in a protected test at that locus [21,22]. Here, following Benjamini & Yekutieli [23], we applied an FDR of 0.10 to identify other indirect effects at a given locus. To further validate loci, we investigated the phenotypic correlations between the directly and indirectly effect loci (see table S1, electronic supplementary material).

**Table 1.** Direct and indirect genetic effects during family interactions. After the locus is identified, the phenotypes affected by the locus are listed with direct effects first, followed by the QTL position in Mb, the 1.5 LOD confidence interval, the genome-wide peak marker likelihood ratio statistic (LRS) and log of the odds (LOD) score and associated *p*-value, the number of genes within the interval, the allele that increases the trait value, and finally the coefficient of determination as obtained from a regression on BXD genotype. All loci have exceeded significance thresholds at the genome-wide level and corrected for multiple traits as outlined in the Material and methods except the indirect effect on B6 maternal activity at *SocInt15*, which narrowly missed the multiple trait threshold but was retained due to the high phenotypic correlation between the direct and indirect effect (electronic supplementary material, table S2).

locus	phenotype	QTL position (Mb)	confidence interval (Mb)	max LRS	max LOD	max *p*	no. genes	allele increasing trait value	coefficient of determination (*R^2^*)
*SocInt2*	BXD sibling solicitation d14	75.857–76.309	73.31–77.355	20.016	4.342	0.045	49	D2	0.590
	B6 maternal suckling d14	74.928–75.515	74.928–75.515	6.426	1.394	0.992	49	D2	0.601
*SocInt4*	BXD sibling sucking d6	11.148–11.507	10.826–13.031	19.672	4.277	0.044	21	B6	0.603
	B6 sibling activity d6	11.148–11.507	10.826–13.031	20.285	4.4	0.030	21	B6	0.603
	B6 sibling sucking d6	11.148–11.507	10.826–13.031	20.652	4.48	0.032	21	B6	0.601
	B6 maternal suckling d6	11.148–11.507	10.826–13.031	19.517	4.234	0.045	21	B6	0.611
*SocInt10*	BXD sibling sucking d14	101.578–102.633	99.07–103.025	23.537	5.117	0.035	17	D2	0.597
	B6 sibling sucking d14	101.578–102.633	99.07–103.025	23.847	5.173	0.029	17	D2	0.590
	B6 maternal suckling d14	101.028	97.112–103.025	19.857	4.307	0.085	27	D2	0.601
*SocInt15*	BXD sibling sucking d14	3.619–4.349	3.229–6.298	14.671	3.189	0.223	19	D2	0.597
	B6 maternal suckling d14	3.619–4.349	3.229–6.298	22.538	4.889	0.036	19	D2	0.601
	B6 sibling sucking d14	3.619–4.349	3.229–6.298	15.058	3.266	0.205	19	D2	0.590
	B6 maternal activity d14	3.619–4.349	3.229–7.273	12.373	2.684	0.551	26	B6	0.670

To identify candidates, we firstly used the ‘Phenotypes, Alleles & Disease Models Search’ (http://www.informatics.jax.org/allele) on Mouse Genome Informatics [24] to find phenotypes associated with each of the genes within the loci we identified. Second, QTLminer [25] was used to summarize information about candidate genes, including if they have non-synonymous SNPs (nsSNPs) or insertions or deletions (indels) in the BXD lines. We note that in the latter case, potential causal variants may be omitted if they are, for example, regulatory variants in unidentified enhancers. Finally, to obtain a broad estimate of heritability we used genotype as a predictor and trait value as a dependent variable in an ANOVA.

## 3. Results and discussion

Four social interaction loci were identified that directly influenced offspring solicitation, sucking, and activity, and, at the same time, indirectly affected sibling and maternal behaviour (table 1); three of which during the weaning period (d14), and one during early lactation (d6). At the phenotypic level, we found significant correlations between directly and indirectly affected traits. We can thus not only show how genetic variation affects the phenotype of a focal individual directly, and indirectly those of social partners, but also how this indirect effect arises as a consequence of genes expressed in another individual at the phenotypic level.

Specifically, three loci on chromosomes 4, 10, and 15 directly affected offspring sucking behaviour either during early lactation (d6) or during the weaning period (d14)*.* All three loci also indirectly influenced the sucking behaviour of their unrelated litter mates, as well as maternal provisioning in their unrelated mothers. At the phenotypic level, directly and indirectly influenced traits were positively correlated. Activity levels were indirectly affected by *SocInt4* in siblings; and in mothers by *SocInt15*, which showed a negative correlation with both solicitation and sucking (electronic supplementary material, table S1). It is important to note that there is no genetic variation among either mothers or litter mates so we do not necessarily expect that the same traits in both half litters are affected by direct and indirect effects. Further, given we expect competition between siblings over resource share, it is surprising to see that unrelated litter mates actually benefited from increased sucking behaviour of their litter mates. Given limited access to maternal teats, we expected that genes increasing sucking behaviours would increase competition and thus reduce sucking in their litter mates. However, our results suggest that the indirect effect on increasing maternal provisioning benefited unrelated littermates who then also increased their sucking behaviour.

Finally, while we have standardized environmental conditions among both BXD and B6 mothers pre- and postnatally as much as possible, it is important to note that differences among B6 half litters may arise for reasons other than genetic differences among BXD genotypes. Firstly, pre-natal maternal (genetic or environmental [26,27]) effects among the B6 mothers may contribute to differences among B6 half litters. Secondly, BXD litters may vary in their experience of B6 mothers or siblings.

### (a) Variation explained by direct and indirect effects

We next sought to establish how important IGE are in explaining variation in behavioural strategies during family interactions. Here, we calculated the proportion of variation explained, either for direct (i.e. variation in BXD phenotype) or for indirect effects (i.e. variation in B6 phenotype). Indeed, a large proportion of variation in behavioural traits could be explained by IGE (table 1). For maternal traits that are indirectly affected by offspring genotype, such as suckling, this ranges from 60% to 67% while for indirectly affected sibling traits, such as activity and solicitation, the range is 56–60%. By comparison, the values for direct genetic effects range from 59% to 62% (table 1). This clearly underlines the significance of indirect effects caused by genetic variation in other family members. For trait evolution, the impact of the significant contribution of indirect effects to trait variation is crucial as they can impose constraints on evolution [2].

### (b) Candidate gene analysis

Our hypothesis was that variation in behavioural traits observed during family interactions are caused by genetic differences between the lines (i.e. genotypes). We used a systems genetics analysis for directly affected traits to identify a list of candidate genes within the loci identified in the mapping analysis. However, we note that as with any mapping study, our loci contain many genes (for an exact number see electronic supplementary material, table S2). While we focused on those candidates that have functional polymorphisms among the BXD genotypes, it may be possible that causal genes are located outside the interval, or affect expression of genes outside the locus.

We begin with *SocInt2* (chromosome 2, 73.310–77.355 Mb). This locus directly affected offspring solicitation on d14, as well as maternal suckling behaviour indirectly. Genes within this locus are linked to early growth and body weight. Twenty of the 49 genes within this locus have nsSNPs or indels among the BXD genotypes and, among those 20, several alter behaviour and weight, such as *Chn1* and *Atf2* [28,29] and *Nfe2l2* [29,30], which are thus the best candidates.

*SocInt4* (chromosome 4, 10.826–13.031 Mb) affected offspring sucking directly, and maternal suckling, sibling activity, and sucking behaviour indirectly during early lactation (day 6). Only 13 genes within the locus have nsSNPs or indels, producing a limited list of potential candidates. A potential candidate is *Dpy19l4*, which contains 18 nsSNPS and three indels, and has been linked to early brain development [31,32]. *Rbm35a* (now known as *Esrp1*) is another candidate, as ablation causes cleft lip and palate [33], and therefore more minor changes may alter the ability to suck.

Next, *SocInt10* (chromosome 10, 97.112–103.025 Mb) influenced pup sucking directly, and sibling sucking and maternal suckling indirectly. This region has been linked to several weight and obesity QTL. Only seven of the 27 genes within the confidence interval have nsSNPs or indels. *Dusp6* is a possible candidate, as it contains three nsSNPs, and is involved in several metabolic pathways, and is important for normal development [34]. *Cep290* is another potential candidate, as mutations in the gene have been linked to slow postnatal weight gain (https://www.jax.org/strain/013702), which may be due to reduced feeding. Mutations in the gene have also been linked to problems with vision, olfaction, and taste [35–37], possibly causing pups to be less responsive to behavioural signals from the mother.

Finally, *SocInt15* (chromosome 15, 3.229–7.273 Mb) affected the level of offspring sucking behaviour directly, and, indirectly, maternal suckling and activity, and sibling sucking during the weaning period on d14. Within the locus are 11 genes with nsSNPs or indels, producing a small number of candidate genes. We find two out of eight possible candidates alter behaviour, *Ghr* and *Sepp1*. *Ghr* influences feeding behaviour and growth [38], while *Sepp1* influences parental behaviour [39] and grooming behaviour [40]. *Sepp1* has nsSNPs and *Ghr* has both nsSNPs and indels.

## 4. Conclusion

The key result of our study is that IGE significantly contribute to explaining differences between family members in behavioural strategies, and are as important as direct genetic effects, with fundamental consequences for trait evolution [2]. We have shown that indirect effects can have counterintuitive effects, for example, in sibling competition whereby litter mates can actually benefit from increased sucking behaviour in a focal individual. This result supports predictions from recent coadaptation models [41], which demonstrated that in family social environments coadaptation accelerates the evolution of cooperation rather than competition. Indeed, our empirical support for these theoretical predictions casts some doubt on the commonly adopted sibling conflict paradigm [7]. Clearly, the dynamics and consequences of selection pressures on behavioural strategies during family interactions are more complex than one might assume. Our study shows that genetic variants involved in different pathways ranging from morphology to behavioural development may affect our traits, and allelic variation at these loci increases or reduces the trait value.

By identifying the direct and indirect sources of genetic variation in key traits during family interactions our study demonstrates that trait variation is not just caused by genes expressed in a focal individual but that such direct effects influence variation in multiple traits in different family members indirectly. Thus, direct effect loci may cause indirect effects either pleiotropically or by being closely linked to variants causing indirect effects. Our results suggest that we need to view behavioural strategies really as the result of selection on genetic variation in all interacting partners.

## Supplementary Material

Supplementary Table 1

## Supplementary Material

Supplementary Table 2

## Data accessibility

Data are available on genenetwork.org using the following record ID numbers: 18781–18790.
